# A system dynamics model for pests and natural enemies interactions

**DOI:** 10.1038/s41598-020-79553-y

**Published:** 2021-01-14

**Authors:** Bonoukpoè Mawuko Sokame, Henri E. Z. Tonnang, Sevgan Subramanian, Anani Y. Bruce, Thomas Dubois, Sunday Ekesi, Paul-André Calatayud

**Affiliations:** 1 International Centre of Insect Physiology and Ecology (icipe), P.O. Box 30772-00100, Nairobi, Kenya; 2 International Maize and Wheat Improvement Center (CIMMYT) ICRAF House, United Nation, Avenue, Gigiri, Village Market, P. O. Box 1041, Nairobi, 00621 Kenya; 3 IRD, CNRS, Université Paris-Saclay, UMR Évolution, Génomes, Comportement et Écologie, 91198 Gif-sur-Yvette, France

**Keywords:** Ecology, Zoology, Mathematics and computing

## Abstract

Stemborers (*Busseola fusca*, *Sesamia calamistis* and *Chilo partellus*), the fall armyworm (*Spodoptera frugiperda*) and associated parasitoids constitute an interacting system in maize fields in Kenya. This work aims at developing and evaluating models that represent the evolution of those interactions by applying system thinking and system dynamics approaches with its archetypes [causal loop diagram (CLD), reinforcing (R) and balancing (B)] to analyse the population of these multi-species systems. The software Vensim PLE 8.0.9 was used to implement the models and carry out the simulations of single- and multi-species systems. The results showed that when a single pest species with its associated parasitoids interact with the host plant, the species was able to establish and sustain by cyclical relationship between populations of the pest and the associated parasitoids. However, in multi- pest species systems, dominance of *S. frugiperda* and *C. partellus* over *B. fusca* and *S. calamistis* was observed, but without extinction. However, there was a likelihood for *B. fusca* being displaced by *C. partellus*. Overall, the models predict the co-existence of fall armyworm with stemborer species as an additional pest of maize in Africa that need to be considered henceforth in designing IPM strategies in maize.

## Introduction

Globally, maize *Zea mays* L. (Poaceae) production was estimated at 1.3 billion tons in 2018^1^. Maize is one of the most important cereal crops in sub-Saharan Africa (SSA)^2^. In Kenya, maize is grown predominantly by smallholder farmers^3^ and constitutes a vital source for household livelihoods^4^. The productivity of maize is affected by a wide array of biotic and abiotic stresses that reduce the quantity and quality of its yields. Insect pest pressure is among the major threats that constrain maize crop from reaching its maximum potential yields. A complex of lepidopteran stemborers and the recent invasive fall armyworm *Spodoptera frugiperda* (J.E. Smith) (Lepidoptera: Noctuidae) are the primary pests of maize crop in many parts of the world, including Kenya, causing yield losses ranged from 30 to 70%^3,5^. These pests are responsible for significant losses of maize upon infestation. Evidence to date suggests that with climate change, these pests are continuing to spread to new areas^6–8^.

In SSA, the noctuid stemborers *Busseola fusca* (Fuller) and *Sesamia calamistis* (Hampson) (Lepidoptera: Noctuidae), and the crambid stemborer *Chilo partellus* (Swinhoe) (Lepidoptera: Crambidae) are economically the most important lepidopteran pest species that severely limit maize productivity as a result of a continuous infestation of the crop throughout its growth stages^9,10^. In maize fields, these stemborers may occur as single species or as a community of mixed species^11,12^. Among these stemborers, *C. partellus* is exotic and invaded eastern Africa in the 1930s^9^. This species has competitively displaced *B. fusca* in the highlands of South Africa^13^. It has also displaced *Chilo orichalcociliellus* Strand (Lepidoptera: Crambidae) in the coastal region of Kenya^14^ and might get an advantage over *S. calamistis* in the utilization of maize in the context of future climate change^6,15^. Recently, *S. frugiperda* invaded SSA, where it seriously limits maize yields^16,17^. Field observations indicated that it interacts strongly with maize stemborer systems^18^ and might also displace them.

Invasive insect herbivores have the prospective to significantly hamper with prevailing insect parasitoids species in invaded areas; this mechanism can occur in different ways: (1) interferences with the volatiles that attract the insect parastoids to unsuitable the host; if the plants can be infested by both the native and invader, the later produces volatiles that are less attractive to parasitoids^19^; and (2) the parasitoids can attempt to parasitize the invasive insect, with low chance to complete their development. This is considered as a waste of time and energy that can negatively affect the fitness of the parasitoids^19,20^. These interferences can therefore have detrimental consequences on a pre-existing biological control process^21^.

Although stemborer species and the fall armyworm have been considered a serious constraint to maize production, few studies have illustrated the interactions among these complexes of pest species. System dynamics, first developed by Forrester^21^, offers a useful method to understand and describe such interactions. That approach, that was originally developed for engineering and administration studies is increasingly been applied to other fields such agriculture, health, economic, and social science^21,22^. The method takes in consideration a set of elements that interact continuously as a component with structure, which undergoes changes^22,23^. The analysis of the system structure (model) by scenarios provides an understand the system behaviour with time. Using differential equations and the Routh–Hurwitz criteria, Mwalusepo et al.^24^ studied the stability of insect species competing for resource. The study revealed that when a species feeds on a resource, the species will be able to establish and sustain a stable population that fluctuates based on the resource availability. However, in a competing context with many species feeding on a single resource, it is observed that the combinations of three parameters (half-saturation, growth rate and mortality rate) determine which species has the upper edge on the resource. In another study, Neill^25^ applied matrix model of the competition coefficients to study the community of species to reveal different patterns of interspecific interactions and estimate the maximum number of interacting species expected in a community. This work therefore aims at developing and evaluating models that represents the interactions of maize stemborer species and *S. frugiperda* populations and their associated parasitoids in a multi-species community in maize fields.

## System components and structure of the models

The stemborers, *B. fusca*, *S. calamistis* and *C. partellus* are the most important pests of maize in Kenya^9^. The three stemborers frequently occur as single or mixed species communities^11,12^ whose structure varies with agro-ecological zones. *Busseola fusca* is generally the dominant species in the highlands, while *C. partellus* dominates in the lowlands^6,26^, and *S. calamistis* occurs at all altitudes^27^. These stemborer species often occur as a mixed community of the three species in the mid-altitudinal regions^7,12^. *Spodoptera frugiperda*, since its first report in the western region of Kenya in 2017, has been confirmed throughout the different agro-ecological zones by the early cropping season in 2018^28^.

Several studies have documented parasitoids associated with the three stemborers in the different agroecological zones^29–31^. In cultivated habitats in Kenya, the most common parasitoids of all three species are the larval parasitoids *Cotesia flavipes* Cameron and *Cotesia sesamiae* (Cameron) (Hymenoptera: Braconidae) followed by the pupal parasitoids *Xanthopimpla stemmator* (Hymenoptera: Ichneumonidae) and *Pediobius furvus* Gahan (Hymenoptera: Eulophidae), and the tachinid *Siphona* sp. (Diptera: Tachinidae)^29–31^. Since its invasion, research for development efforts has highlighted the effectiveness of several integrated pest management strategies for *S. frugiperda*, including new association of indigenous natural enemies with *S. frugiperda* such as the larval parasitoids *Cotesia icipe* Fernandez‐Triana & Fiaboe (Hymenoptera: Braconidae)*, Charops* sp. Holmgren (Hymenoptera: Ichneumonidae) , *Coccigydium luteum* Brullé (Hymenoptera: Braconidae)*, Palexorista zonata* Curran (Diptera: Tachinidae); the egg-larval parasitoid *Chelonus curvimaculatus* Szépligeti (Hymenoptera: Braconidae) and the egg parasitoids *Telenomus remus* Dixon (Hymenoptera: Platygastridae) and *Trichogramma chilonis* Ishii (Hymenoptera: Trichogrammatidae)^28,32^.

*Busseola fusca* and *S. calamistis* females deposit the eggs between the leaf sheath and the stem of plant as a protection strategy against the environment and the natural enemies, whereas *C. partellus* and *S. frugiperda* females deposit eggs directly on leaf surfaces^33,34^. Upon emergence, the young larvae are dispersed by ballooning while older larvae disperse by crawling, resulting in a redistribution of the insect infestations within and between plants in maize fields^35^. The stemborer larvae feed on young leaves until the third instar and later bore into maize stems. *Spodoptera frugiperda* larvae feed only on leaves during their whole development, especially the central leaves in the plant whorl^36,37^. In addition, in maize fields at tasseling stage, *S. frugiperda* larvae can be found feeding on the tassels and subsequently on the ear, silk, cob and even in stemborer's holes^36,38^. Therefore, *S. frugiperda* and stemborer larvae may interact by sharing the same niche at young developmental stages and even when the stemborer larvae migrate from the leaves to stems.

The four pest species (three stemborers + *S. frugiperda*), in addition to their associated parasitoids and the maize plants that serve as the resource for the pests, constitute the system under study. Several cases are considered because these insects occur at different spatial distributions aross different agroecological zones. The analyses were subdivided in four cases: (1) a single pest species feeding on maize plants and its parasitoids, (2) two species competing on maize plants and their parasitoids, (3) three species competing on maize plants and their parasitoids, and (4) four pest species together on maize plants and their parasitoids.

## Results

### One pest species and its parasitoids and the resource (maize plants)

**Figure 1:**
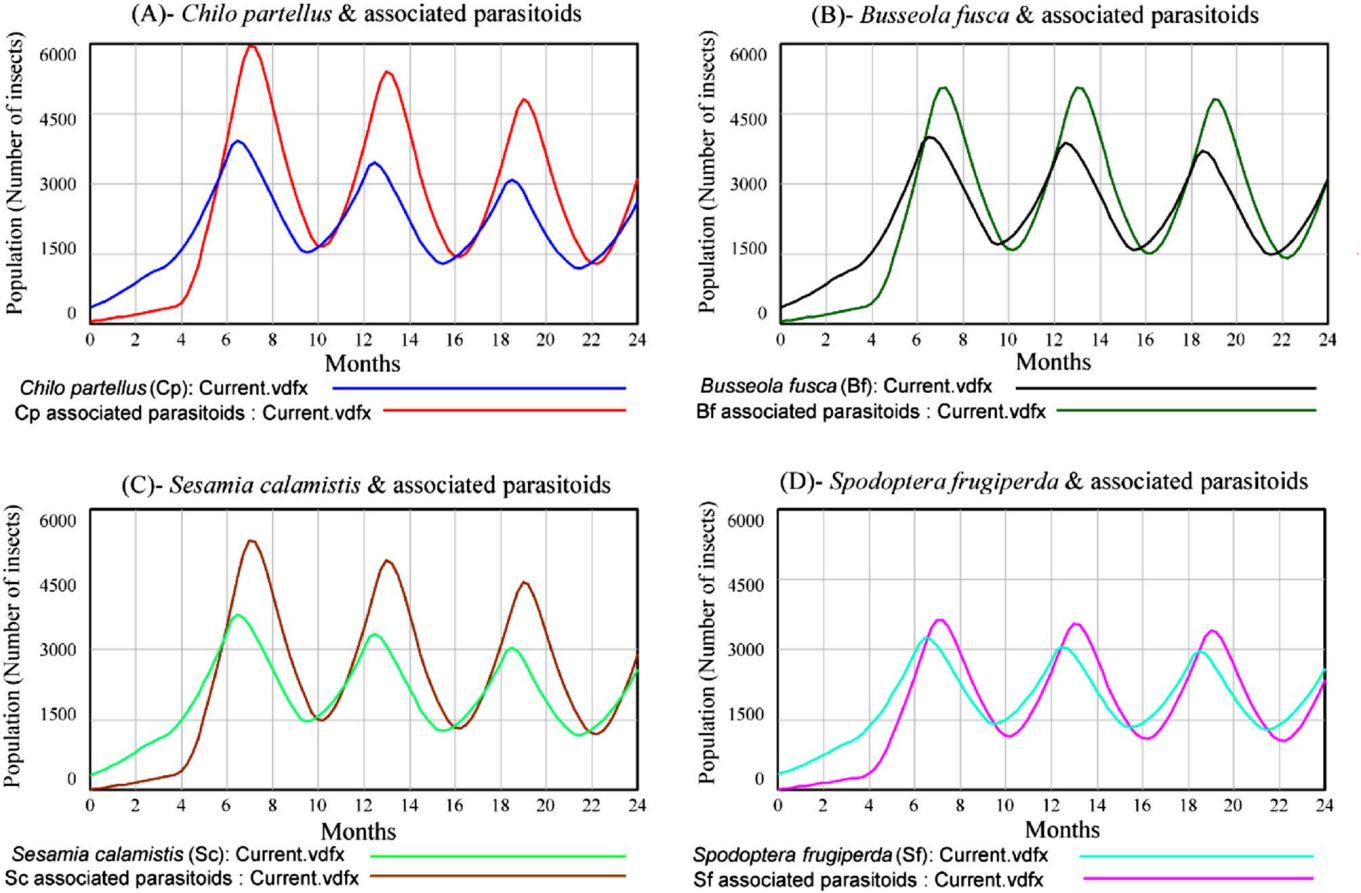
Populations of *Chilo partellus* (**A**), *Busseola fusca* (**B**), *Sesamia calamistis* (**C**) and *Spodoptera frugiperda* (**D**) as a single pest species with their respective associated parasitoids in the system.

For each pest species and its associated parasitoid populations, the outcomes of the models showed that both populations marginally increased at the beginning. After 4 months, the relationship became cyclical between a host (pest) and its associated parasitoids (Fig. 1A–D). As the population of parasitoids increased, the pest population decreased, which in turn caused parasitoids population to decrease. As parasitoids population decreased, the pest population was able to recover, and its population increased. Subsequently, the parasitoids population increased and the cycle began again. The three stemborer species had similar populations with a maiximum peak after 6 months of about 4900 individuals (Fig. 1A–C) while the maximum peak of *S. frugiperda* poplulation (Fig. 1D) was 17% less than those of stemborers*.* The peaks of the populations of parasitoids of the three stemborers species reach a level above 4500 individuals after 7 months while the population of parasitoids associated with *S. frugiperda* was below 4000 individuals during the same period (Fig. 1A–D).

### Two pest species and associated parasitoids and the resource (maize plants)

**Figure 2:**
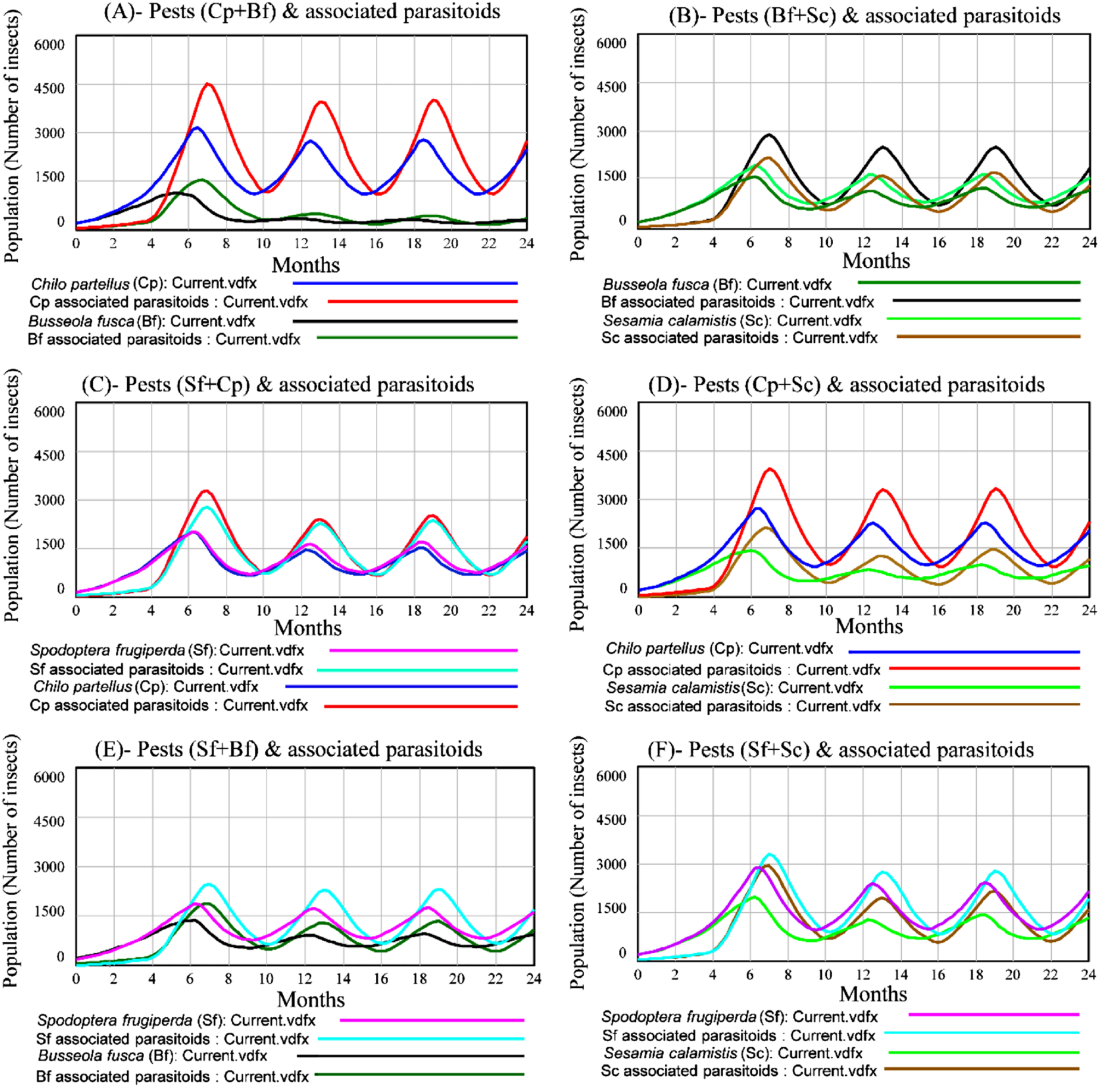
Populations of *Chilo partellus*, *Busseola fusca*, *Sesamia calamistis* and *Spodoptera frugiperda* and their associated parasitoids in two multi- pest species competitive systems. (**A**) *C. partellus* + *B. fusca* (Cp + Bf) system; (**B**) *B. fusca* + *S. calamistis* (Bf + Sc) system; (**C**) *S. frugiperda* + *C. partellus* (Sf + Cp) system; (**D**) *C. partellus* + *S. calamistis* (Cp + Sc) system; (**E**) *S. frugiperda* + *B. fusca* (Sf + Bf) system; (**F**) *S. frugiperda* + *S. calamistis* (Sf + Sc) system.

In a two species system, a strong unilateral competitive interaction was revealed in the *C. partellus* and *B. fusca* system (Fig. 2A) representing 81.18% and 18.82%, respectively of the total pest populations in the system. The population of *B. fusca* was largely outcompeted by *C. partellus* after 6 months and its population was drastically reduced in a two species system as compared to when it was the sole species in the system. In the system of either *B. fusca* and *S. calamistis* (Fig. 2B) or *C. partellus* and *S. frugiperda* (Fig. 2C), bilateral competitive interactions were strong, leading to the decline of both the species populations but without dominance. Although bilateral competitive interactions were revealed in other two multi- pest species systems, *C. partellus* was most prevalent to *S. calamistis* (Fig. 2D) representing 66.08% and 33.92%, respectively and *S. frugiperda* was most prevalent to *B. fusca* (Fig. 2E) and *S. calamistis* (Fig. 2F) after 6 months and represented 60.53% and 39.47%, respectively of the total pest populations in the system. In addition, the model showed that the populations of each pest species in two pest species systems (Fig. 2A–F) declined as compared to those in sole pest species systems (Fig. 1A–D). However, the average total pest populations (population size of each pest) in sole-pest species systems represented only 85.36% of total average pest populations in two-pest species systems (population size of any given combination of two pest species). In each combination, the associated parasitoid populations proportionally varied with their respective host population fluctuation as parasitoid population tracked the peaks of the pest population.

### Three and four pest species and associated parasitoids and the resource (maize plants)

**Figure 3:**
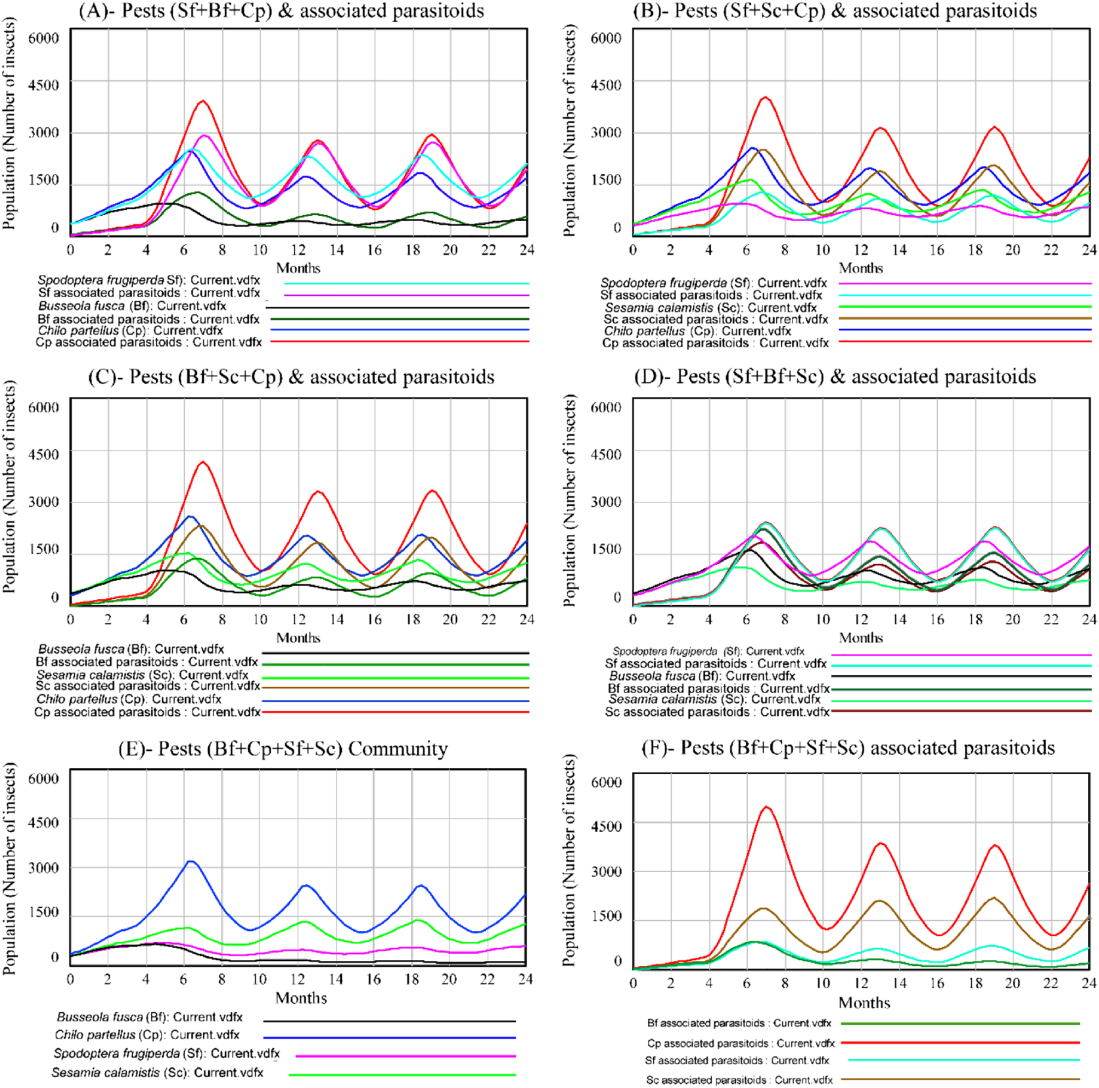
Populations of *Chilo partellus*, *Busseola fusca*, *Sesamia calamistis* and *Spodoptera frugiperda* and their associated parasitoids in three and four multi-species competitive systems. Three multi-species: (**A**) *S. frugiperda* + *B. fusca* + *C. partellus* (Sf + Bf + Cp); (**B**) *S. frugiperda* + *S. calamistis* + *C. partellus* (Sf + Sc + Cp); (**C**) *B. fusca* + *S. calamistis* + *C. partellus* (Bf + Sc + Cp) and (**D**) *S. frugiperda* + *B. fusca* + *S. calamistis* (Sf + Bf + Sc) systems. Four multi-species: (**E**) *B. fusca* + *C. partellus* + *S. calamistis* + *S. frugiperda* (Bf + Cp + Sc + Sf) and (**F**) their associated parasitoids systems.

In three species systems after 6 months, *C. partellus* and *S. frugiperda* co-exist representing 45.45% and 40.42% of the total pest populations in the system but competitively dominated *B. fusca* population that represented only 14.13% (Fig. 3A). However, in *C. partellus* + *S. calamistis* + *S. frugiperda* three species system, *S. calamistis* became dominant over *S. frugiperda* (Fig. 3B). They represented 44.80%, 31.43% and 23.77% , respectively of the total pest populations in the system. The system of the three stemborer species (Fig. 3C) showed the dominance of *C. partellus* (47.93%) followed by *S. calamistis* (31.70%) and *B. fusca*(20.37%), respectively. *Spodoptera frugiperda* was the dominant species followed by *B. fusca* and *S. calamistis* in three pest species system (Fig. 3D). In each system, the parasitoid population fluctuation evolved according to its host population fluctuation. Furthermore, the model showed that the population of each pest species in three pest species systems (Fig. 3A–D) declined as compared to those in two pest species systems (Fig. 2A–F). However, the average total pest populations in two-pest species systems represented only 70.07% of total average pest populations in three-pest species systems.

In four pest species system, *C. partellus* was the dominant species (48.79%), followed by *S. calamistis* (28.34%), *S. frugiperda* (14.85%) and *B. fusca* (8.02%), respectively (Fig. 3E), with the same trend of their associated parasitoids (Fig. 3F). Comparing four pest species system populations to those in three pest species systems, the population of each pest species declined except that of *C. partellus*. The total average pest populations in three-pest species systems represented only 89.88% of those in four pest species system.

## Discussion

In this study, we modeled the dynamics and interactions of populations of three maize stemborer species and the fall armyworm, as well as their associated parasitoids, in either single or multi-species systems. The population dynamics of single pest species systems presented S-shaped growth with overshoot logistic form of the well-known Lotka–Volterra prey predator system^39,40^. The S-shaped form could be due to the negative feedback in the loop diagram that slowly limits the growth as the growth rate reaches the limit. However, the negative feedback contains time delays due to the variability of available resources (host plants) leading to intra-specific competitive interaction that affected the abundance of the pest. The time delay in the negative feedback causes the system to exceed the limit value and exhibit oscillation behavior around the limit value as previously reported by Sterman^41^. Furthermore, the presence of parasitoids influences the host population dynamics leading to a cyclical relationship between a host (pest) and its associated parasitoids as previously demonstrated by the Lotka-Volterra predator–prey model function^39,40^. For each of the three stemborer species systems, the associated parasitoids populations grew faster than their respective host populations. Din and Donchev^42^ reported that in a host-parasite interaction, if a host population is a pest, then according to the Leslie–Gower model, a fast-growing parasite population with a growth rate larger than that of the host significantly reduces the host population. Therefore, the present model indicates a significant effect of parasitoid on stemborers population regulation. Despite high outbreaks reported for *S. frugiperda*, its population dynamics in our single-species system was actually the lowest which might be due to its high cannibalism rate reported in the literature^43,44^ and also because the high outbreaks across SSA was reported during the first months of infestation when equilibrium was not yet established; whereas stemborers have been present for decades.

Within stemborer species in multi-species systems (either in two or three species systems), *C. partellus* exhibited dominance whenever involved in a system. Several previous studies have reported the dominance of *C. partellus* over *B. fusca* and *S. calamistis* when they co-exist^15,19,24^. In two-species system with *C. partellus* and *B. fuca*, the model showed that *C. partellus* has displaced *B. fusca* population with time. Those competitive interactions may justify the spatial distribution of these stemborer species in Kenya. Previous studies have reported that *B. fusca* and *S. calamistis* co-exist in the highlands with dominance of *B. fusca* species, while *C. partellus* and *S. calamistis* co-habituate in the lowlands with dominance of *C. partellus*^6,25,26^. These three stemborer species were reported to occur as a mixed system in the mid-altitudinal regions with dominance of *C. partellus*^7,12^. Furthermore, studies in South Africa showed that *C. partellus* has expanded its distribution into highland region and has competitively displaced *B. fusca* population in that area^13^.

On the other hand, the model demonstrated that bilateral competitive interactions were strong in *B. fusca* + *S. calamistis* and *C. partellus* + *S. frugiperda* two-species systems where both species populations fluctuate and dropped considerably. Those behaviours may be explained by the overlapped ecological niches of these pest species. The females of *B. fusca* and *S. calamistis* deposit the eggs between the leaf sheath and the stem of plant, whereas *C. partellus* and *S. frugiperda* deposit eggs directly on leaf surfaces^33,34^. Therefore, the interactions in those systems might start at neonate stage when the eggs hatched or even at egg stage by sharing the same ecological niches. Sokame et al.^35^ have also demonstrated that the larvae of pest species in those respective systems shared the same behaviour in terms of ballooning and crawling. All those common life traits might lead them to strong competition. Zhou et al^45^ have demonstrated that in the nature, species that are living together in the same or similar niches because they have one or several kinds of similar behaviours are highly competitive. The dominance of *S. frugiperda* species in the system of either *B. fusca* or *S. calamistis* in two species systems or with both species in three species system in the present model might be the intraguild predation preference of *S. frugiperda* to the detriment of cannibalism in interspecific systems which is reflected in competition coefficients of *S. frugiperda* in multi-species systems used in our study. Bentivenha et al.^46^ demonstrated that *S. frugiperda* in multi-species system with *Helicoverpa zea* (Boddie) (Lepidoptera: Noctuidae), its cannibalism decreases to the detriment of intraguild predation of the other species.

Overall, the outputs of the simulations indicated that *C. partellus* and *S. frugiperda* species were competitively superiors over *B. fusca* and *S. calamistis*. However, in three or four multi-species involving *C. partellus* with *S. calamistis* and *S. frugiperda*, *S. calamistis* took the advantage and dominated *S. frugiperda*. *Chilo partellus* and *S. frugiperda* are exotic species in Africa while *B. fusca* and *S. calamistis* are indigenous. Invasive insect pest species have the potential to rapidly establish and spread to new areas^47^. The organisms that arrive and establish themselves in a new range of hosts are positioned to have adverse effects on the surrounding fauna and also results in the extinction of other species^48^. They often affect native species populations and systems by competing for the same resource^49^. For example, the Asian adelgid, *Pineus boemeri* Annand, has been shown to be competitively superior and to displace a native congener, *P. coloradensis* (Gilette) in red pine (*Pinus resinosa* Aiton) plantation in Eastern USA, possibly through the reduction of host plant quality and forcing *P. coloradensisto* to less suitable sites^50^. The invasive fruit fly *Bactrocera invadens* Drew, Tsuruta & White (Diptera: Tephritidae), has displaced the indigenous mango fruit fly, *Ceratitis cosyra* Walker (Diptera: Tephritidae) 4 years after invasion in Kenya and become the dominant fruit fly pest of mango^51^. Temperature and resource pre-emption were demonstrated to be key factors contributing to the competitive success of the invasive fruit fly *B. invadens* over the indigenous mango fruit fly, *C. cosyra* in Kenya^52^. Fabre et al.^53^ demonstrated a form of resource competition between native and exotic seed chalcids, *Megastigmus* spp. and displacement of the native species. Similarly, the African stemborer *B. fusca* seems to have been displaced from sorghum fields by the Asian invasive stemborer *C. partellus*^13^ possibly due to deterrence of the native species by the invasions or due to differences in host plant phenology.

The comparison of sole pest species systems with multi-pest species systems of the models showed that the population of each species declined in multi-pest species systems and more the number of pest species involved in the system increased more the population of each species declined. In contrary, the total number of pest populations in the systems increased with the number of pest species involved in the system. Therefore, the reduction of stemborer populations in maize fields with the arrival of *S. frugiperda* was even overtaken up by that latter. Those results indicate the overall pest abundance increasing in maize fields with the invasion of fall armyworm in maize stemborer systems with more infestations and damages, leading to the increasing of the smallholder incomes losses in maize production. However, the fact that no competition between parasitoid species was considered might have effect on the system dynamics model.

In conclusion, the present models predict the co-existence of *S. frugiperda* with stemborer species in maize fields. *Spodoptera frugiperda* and *C. partellus* dominate over *B. fusca* and *S. calamistis* but without extinction, except that *B. fusca* seems to be displaced by *C. partellus*. Therefore, the invasion of *S. frugiperda* in maize fields in Africa constitutes an additional pest to maize crop that need to be considered within the context of integrated pest management strategy. However, the underpinning mechanisms surrounding the co-existence and possible displacement of other species warrant additional studies.

## Methods

### Modelling and simulations assumptions

To develop the model, the following assumptions have been made:

1. Data obtained under laboratory conditions were used to reproduce and simulate what may occur under field conditions.
2. The growth of insect pest species is limited by a single maize resource, and the parasitoids only depend on their host pests for survival.
3. During the non-cropping season, only 10% of the pest found in maize fields survived on alternative host plants and will give rise to a new pest population in maize field during the subsequent cropping season.
4. A 3-month maize variety (Duma 43, Kenya Seed Company, Nairobi, Kenya) was considered to be used and grown from April to June and from October to December, periods corresponding to the yearly cropping seasons in Kenya.
5. Insect pests were recorded for the first time in the maize field 1 month after planting date.
6. Pest population growth is assumed to follow the Lotka–Volterra competition function^39^.
7. The parasitism level of a given pest species is recorded from the second generation of the pest, thus with an average of 2 months of delay after maize planting.
8. The parasitism level of all parasitoid species on a given host were lumped together and no competition between parasitoid species was taken into account.
9. Simulations were carried out assuming that each system was at the equilibrium state.

### Models simulations and data sources

Before the simulations, a multiple regression procedure was conducted using the R software version 3.5.1 (R Foundation for Statistical Computing, Vienna, Austria) with experimental data from Sokame^56^ on density-dependent of species interaction in laboratory conditions to estimate the competition coefficients of each studied case of species combination as presented in Table 1.

**Table 1:** Vice versa competition coefficients of two, three and four multi-species systems.

Two multi-species combinations								
Bf and Sc			Bf and Cp			Sc and Cp		
j/i	Bf	Sc	j/i	Bf	Cp	j/i	Sc	Cp
Bf	1	2.51e−4	Bf	1	9.12e−5	Sc	1	1.42e−4
Sc	3.31e−4	1	Cp	4.15e−4	1	Cp	3.13e−4	1

Sf and Bf			Sf and Sc			Sf and Cp		
j/i	Sf	Bf	j/i	Sf	Sc	j/i	Sf	Cp
Sf	1	4.1 e−4	Sf	1	1.99e−4	Sf	1	2.2e−4
Bf	3.02e−4	1	Sc	1.07e−4	1	Cp	2.1e−4	1

Three multi-species combinations								
Bf and Sc and Cp				Sf and Bf and Sc				
j/i	Bf	Sc	Cp	j/i	Sf	Bf	Sc	
Bf	1	9e−5	5.1e−5	Sf	1	3.02e−4	3.1e−4	
Sc	8.2e−5	1	1.02e−4	Bf	9.7e−5	1	1.13e−4	
Cp	3.5e−4	2.01e−4	1	Sc	1.02e−4	1.18e−5	1	

Sf and Bf and Cp				Sf and Sc andCp				
j/i	Sf	Bf	Cp	j/i	Sf	Sc	Cp	
Sf	1	1.7e−4	9.2e−5	Sf	1	5e−5	8.7e−5	
Bf	7.6e−5	1	5.7e−5	Sc	2.51e−4	1	1.02e−4	
Cp	1.91e−5	3.09e−4	1	Cp	1.78e−4	2.15e−4	1	

Four multi-species combinations								
Sf and Bf and Sc and Cp								
j/i	Sf	Bf	Sc	Cp				
Sf	1	2.19e−4	2.5e−4	5.1e−5				
Bf	2.4e−4	1	2.07e−4	2.3e−5				
Sc	2.2e−4	3.04e−4	1	2.1e−5				
Cp	2.01e−4	2.02e−4	8e−05	1				

Bf, *Busseola fusca*; Cp, *Chilo partellus*; Sc, *Sesamia calamistis*; Sf, *Spodoptera frugiperda.*

In addition, the parasitism level of all parasitoids species on a given host that were lumped together and other constants in Table 2 were used for the model simulations. Units and models’ commodities were well checked prior to the simulations.

**Table 2:** Value of constants used in model simulation.

Constant	Value	Source/comment
*Chilo partellus* reference fractional parasitism rate*	0.3	Mailafiya et al.^29,30^
*Busseola fusca* reference fractional parasitism rate*	0.25	Mailafiya et al.^29,30^
*Sesamia calamistis* reference fractional parasitism rate*	0.28	Mailafiya et al. ^29,30^
*Spodoperta frugiperda* reference fractional parasitism rate*	0.22	Sisay et al.^28,32^
*Chilo partellus* reference fraction growth rate	0.83	Kroschel et al.^58^
*Busseola fusca* reference fraction growth rate	0.8	Kroschel et al.^58^
*Sesamia calamistis* reference fraction growth rate	0.8	Kroschel et al.^58^
*Spodoptera frugiperda* reference fraction growth rate	0.7	Prasanna et al.^59^
Reference fractional parasitism rate in wild habitat during non-cropping seasons	0.05	5% was considered for each species
Carrying capacity of 1 ha of maize field	62,500	(0.4 × 0.8 m^2^) = 31250 plts/ha × 2/plt
Carrying capacity of 1 ha in non-cropping seasons	625	10%
Maize cropping seasons of a year (April–June and October–December)	3-month of 2 seasons	Sokame et al.^60^
Non-cropping seasons periods of a year (January–March and July–September)	3-month of 2 seasons	Sokame et al.^60^
Number of parasitoids per host	5	Since there are gregarious and solitary parasitoids, the average is settled at 5
Reference pest density	2/0.5	2/plant in maize field and 0.5/plant in wild habitat and same for all pest species
Host-parasitoid meeting probability	3.5%	Same for all pest species
Parasitoid fractional decrease rate	0.7	Same for all pest species
Parasitoid sex ratio	0.46	Obonyo (Pers. Obs.)
Time step	0.25 month	Weekly recording data
Period of the simulation	24 months	The time to get the stability of the model

* The parasitism level of all parasitoids species on a given host were lumped together.

The models were implemented and simulated using the Vensim PLE 8.0.9 platform (Ventana Systems, Harvard, USA), which consists of a graphical environment that permits users to draw the CLD, stocks and flows diagrams and carry out simulations^55^. The dynamics of pest and associated parasitoids populations were considered as stocks and the in/out flows were defined. The inflows were composed of pest or parasitoid population growth rates while the outflows were represented by decrease rates of the pests that have been parasitized or the parasitoids that have completed their life cycle.

As mentioned in the assumptions section, all simulations were conducted at the equilibrium state of each system that is characterized by:

1 $${K}_{i}={N}_{i}+\sum_{j\ne i}^{m}{a}_{ij}{N}_{j}$$

where *Ki* is the column vector of the total number of larvae survived, *Ni* is the column vector of the total number of survived larvae of a given species, and *aijNj* is the “system matrix’’ of the interaction coefficients.

$$\left[ {\begin{array}{*{20}c} {{\text{K}}_{{1}} } \\ {{\text{K}}_{{2}} } \\ {{\text{K}}_{{3}} } \\ \vdots \\ {{\text{K}}_{{\text{m}}} } \\ \end{array} } \right] = \left[ {\begin{array}{*{20}c} {1} & {{\text{a}}_{{{12}}} } & {{\text{a}}_{{{13}}} } & \ldots & {{\text{a}}_{{{\text{1m}}}} } \\ {{\text{a}}_{21} } & 1 & {{\text{a}}_{{{23}}} } & \ldots & {{\text{a}}_{{{\text{2m}}}} } \\ {{\text{a}}_{{{31}}} } & {{\text{a}}_{{{32}}} } & {1} & \ldots & {{\text{a}}_{{{\text{3m}}}} } \\ \vdots & \vdots & \vdots & \vdots & \vdots \\ {{\text{a}}_{{{\text{m1}}}} } & {{\text{a}}_{{{\text{m2}}}} } & {{\text{a}}_{{{\text{m3}}}} } & \ldots & 1 \\ \end{array} } \right] \times \left[ {\begin{array}{*{20}c} {{\text{N}}_{{1}} } \\ {{\text{N}}_{{2}} } \\ {{\text{N}}_{{3}} } \\ \vdots \\ {{\text{N}}_{{({\text{m}} - {1})}} } \\ \end{array} } \right]$$

The absolute value of any (a) reflects the intensity of the interaction on a given species. The system matrix therefore characterizes the first order (linear) relationship of each species with each other in the system.

$$\left[ {\begin{array}{*{20}c} {{\text{K}}_{{1}} } & = & {{\text{N}}_{{1}} } & + & {{\text{a}}_{{{12}}} } & \times & {{\text{N}}_{{2}} } & + & {{\text{a}}_{{{13}}} } & \times & {{\text{N}}_{{3}} } & + & \cdots & + & {{\text{a}}_{{{\text{1m}}}} } & \times & {{\text{N}}_{{\text{m}}} } \\ {{\text{K}}_{{2}} } & = & {{\text{N}}_{2} } & + & {{\text{a}}_{21} } & \times & {{\text{N}}_{1} } & + & {{\text{a}}_{{{23}}} } & \times & {{\text{N}}_{2} } & + & \cdots & + & {{\text{a}}_{{{\text{2m}}}} } & \times & {{\text{N}}_{{\text{m}}} } \\ {{\text{K}}_{{3}} } & = & {{\text{N}}_{{3}} } & + & {{\text{a}}_{{{31}}} } & \times & {{\text{N}}_{1} } & + & {{\text{a}}_{{{32}}} } & \times & {{\text{N}}_{2} } & + & \cdots & + & {{\text{a}}_{{{\text{3m}}}} } & \times & {{\text{N}}_{{\text{m}}} } \\ \vdots & \vdots & \vdots & \vdots & \vdots & \vdots & \vdots & \vdots & \vdots & \vdots & \vdots & \vdots & \vdots & \vdots & \vdots & \vdots & \vdots \\ {{\text{K}}_{{\text{m}}} } & = & {{\text{N}}_{{\text{m}}} } & + & {{\text{a}}_{{{\text{m1}}}} } & \times & {{\text{N}}_{{\text{m}}} } & + & {{\text{a}}_{{{\text{m2}}}} } & \times & {{\text{N}}_{{\text{m}}} } & + & \cdots & + & {{\text{a}}_{{{\text{m}}({\text{m}} - {1})}} } & \times & {{\text{N}}_{{({\text{m}} - {1})}} } \\ \end{array} } \right.$$

### Models formulation

Ordinary differential equation developed to study species population dynamics under competitions were used in this study. All the models used have the generic formulation displayed in Eq. (2). Considering *N*(*t*) as a state variable to denote the insect population abundance at time *t*; the population growth for the *ith* species is defined with the Lotka–Volterra competition equations, which was later modified by MacArthur and Levins^54^ as:

2 $$\frac{{dN_{i} }}{dt} = \frac{{r_{i} N_{i} }}{{K_{i} }}\left( {K_{i} - N_{i} - \mathop \sum \limits_{j \ne i}^{m} a_{ij} N_{j} } \right)$$

where the *N*_*i*_ is the species abundance, *r*_*i*_ is the intrinsic rate of population natural increase, *K*_*i*_ is the species carrying capacity (the maximum attainable population size), *m* is the number of pest species in the system, and *a*_*ij*_ is the effect that an individual species characterized by *jth* can cause to another species characterized with *ith*. The translation of this generic mathematical expression was applied to formulate the equations used to simulate each case studied. The model expressions can be found in Supplementary Appendix.

### Models implementation

The methodology here is rooted in system thinking approach with its archetypes [causal loop diagram (CLD), reinforcing (R) and balancing (B)] by a mental and holistic conceptual framework used to map how the variables, issues and processes are influencing each other in the complex competitions and interactions among and between insect species and the impacts. Although these archetypes are qualitative in nature, they help to disclose and elucidate the fundamental feedback configurations that occur in maize fields when insect pests are competing for resource and associated parasitoids are hunting for hosts. The CLD obtained were converted into a dynamic modelling using stocks, flows, auxiliary links and clouds; which in turn were translated into coupled differential equations for simulations.

#### One pest species and associated parasitoids and the resource (maize plants)

The diagram of causalities represents the basic structure of the system of a given pest species with its associated parasitoids, where arrows show the cause–effect relations. A positive sign indicates direct proportionality of cause and effect, and the negative sign indicates a relation of inverse proportionality. The system is characterized by two negative feedbacks (Fig. 1, Loops B1 and B2) and one positive feedbacks (Fig. 1A, Loop R1) leading to three main relationships:

- as the resource (maize plants) increases, the growth of the pest increases to occupy the available resource resulting in the pest population increase;
- as host availability increases, the probability that the parasitoid encounters its host increases, resulting in higher parasitism, increased host mortality rate and decreased pest population;
- as host mortality rate increases, parasitoid growth rate increases, and parasitoid population increases.

**Figure 4:**
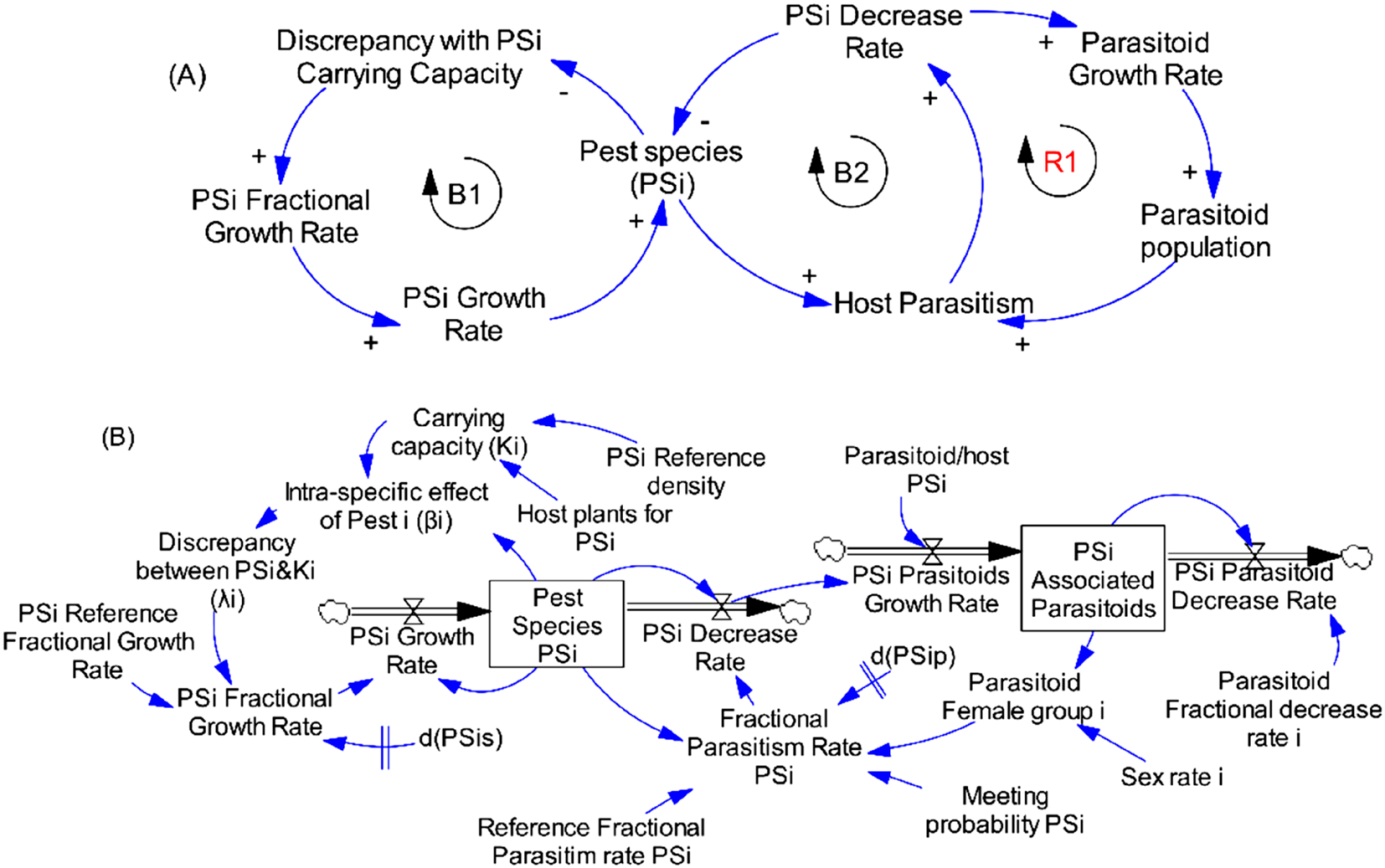
Causal loops (**A**) and flow diagram (**B**) of one pest species and its parasitoids and the resource in the system.

Figure 4B showed the stocks and flows diagram and auxiliary variables obtained from causal loop diagram displayed in Fig. 4A. The single pest species (PSi) and associated parasitoids are the stocks in the system, representing the population size of pest species and parasitoids, respectively, at a given point in time. The growth rates represented the inflows while the decrease rates represented the outflows of the diagram. The auxiliary as well as constant variables that drive the behaviour of the system were connected using information arrows within them and to flows and stocks to represent the relations among variables in terms of equations.

#### Two pest species and associated parasitoids and the resource (maize plants)

**Figure 5:**
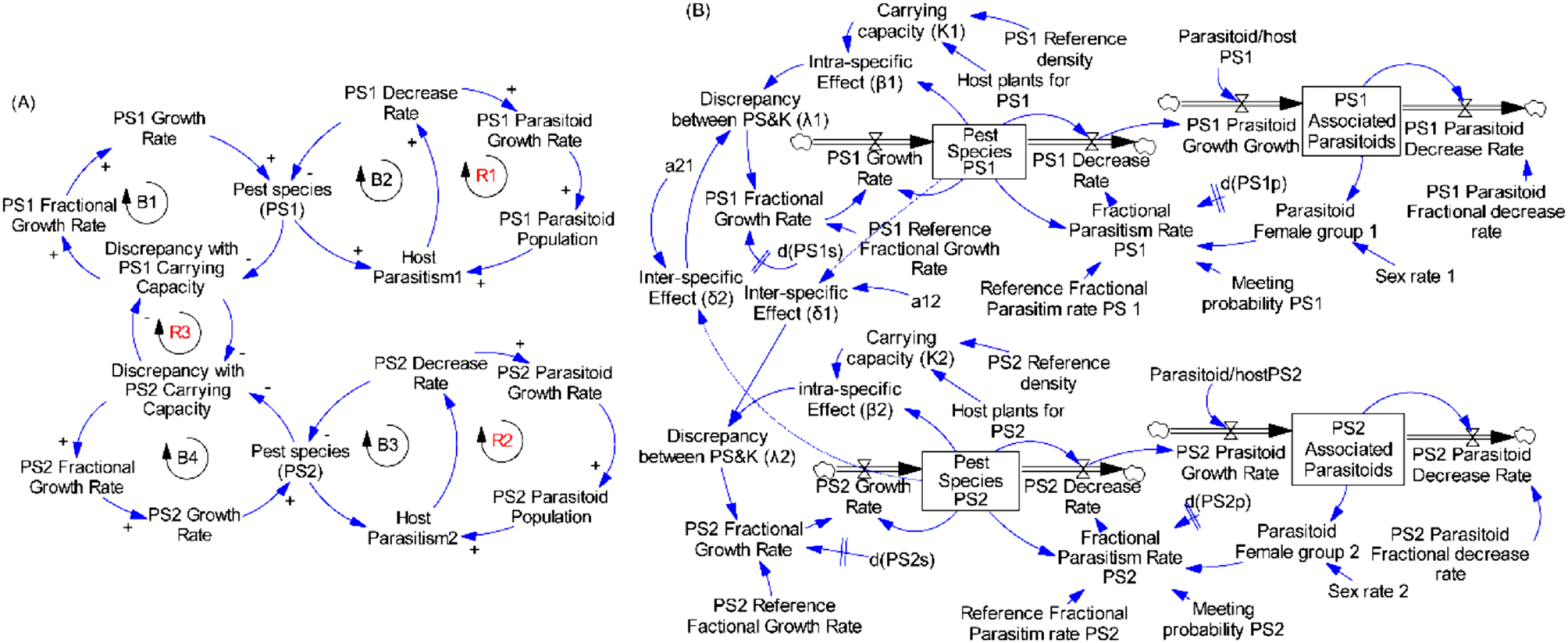
Causal loops (**A**) and flow diagram (**B**) of two pest species and its parasitoids and the resource in the system.

In two multi-pest species system (Fig. 5A), the three previous relationships intervened for each species (Fig. 5A, Loops B1, B2, and R1 for species 1 and Loops B3, B4, and R2 for species 2). In addition, relationship (d: Loop R3, Fig. 5A) described inter-specific competition effect of involved pest species on each other. The stocks and flows diagram of each of the two species occurred with level of discrepancy between the carrying capacity (*K*) and the population size, which additionally is dependent on the intra and inter-specific competition and interations among and between these species (Fig. 5B).

#### Three pest species and associated parasitoids and the resource (maize plants)

**Figure 6:**
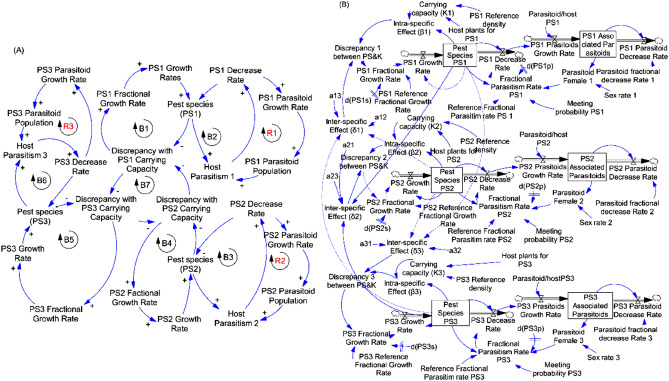
Causal loops (**A**) and flow diagram (**B**) of three pest species and its parasitoids and the resource in the system.

In three multi-pest species system and associated parasitoids system (Fig. 6A), we have the three relationships (a, b and c) previously described for each species (Loops B1, B2, and R1 for species 1; Loops B3, B4, and R2 for species 2 and Loops B5, B6, and R3 for species 3). The relationship (e) represented the inter-specific competitive influence exercised by each of the three species on each other (Fig. 6A,B7). The stocks and flows diagram of individual species in the system occurred with a level of discrepancy between the carrying capacity (*K*) and the population size, which additionally is dependent on the intra- and inter-specific competition and interations among and between these species (Fig. 6B).

**Figure 7:**
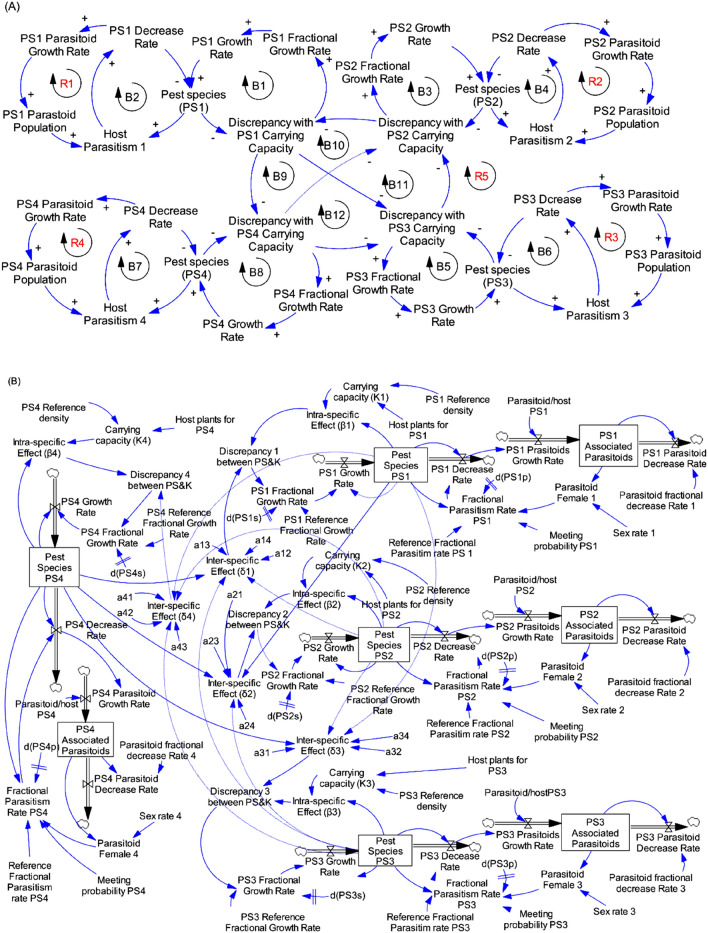
Causal loops (**A**) and flow diagram (**B**) of four pest species and its parasitoids and the resource in the system.

#### Four pest species and associated parasitoids and the resource (maize plants)

In four multi-pest species system (Fig. 7A), the relationships (a, b and c) existed for each of the four species (species 1: Loops B1, B2, and R1; species 2: Loops B3, B4, and R2; species 3: Loops B5, B6, and R3 and species 4: Loops B7, B8, and R4). The relationships (f) represented by the Loops B9, B10, B11 and R5 showed interaction relationships between the four species. The stocks and flows diagram obtained from the system made by four species occurred with a level of discrepancy between the carrying capacity (*K*) and the population size of individual species, which additionally is dependent on the intra and inter-specific competition and interations among and between these species (Fig. 7B).

### Models evaluation

**Figure 8:**
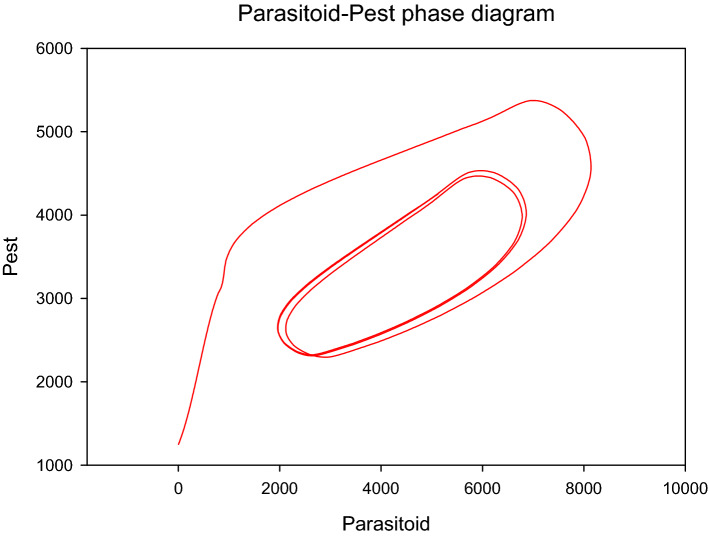
Parasitoid-host phase diagram.

The model equations used in this study are standard with Lotka–Volterra like formulation. These equations have been used by others authors^39,57^ to study biological species involving multiple species interactions. Lack of adequate time series data did not allow to use the conventional evaluation technique that consists of plotting the observed data and simulated outputs for measuring the goodness fit of the models. However, we leveraged on the popularity and enormous amount of studies produced from the famous Lotka Volterra competitive equations. To measure the model’s performance and ensure that the obtained outputs are authentic, we compiled the population densities of all pest species (stemborers and *S. frugiperda*) and parasitoids to establish an interacting system with only two species (pest and parasitoids). The obtained new model was simulated, and the parasitoid-host phase diagram is presented in Fig. 8. The two species competition models produced circular isoclines as phase diagram, as revealed by Lotka–Volterra equations therefore, we concluded the validity of the developed models using stock and flow diagram. The curve of the graph is identical to shape found in literature^39,40^ and used to describe the co-evolution of two species under interactions.

## Supplementary Information

Supplementary Information
