# Supplementary material for: A system dynamics model for pests and natural enemies interactions
Source: Sci Rep. 2021 Jan 14;11:1401. doi: 10.1038/s41598-020-79553-y (PMC7809103; doi:10.1038/s41598-020-79553-y)
Supplement: Supplementary file 1 — Supplementary Information [file 41598_2020_79553_MOESM1_ESM.docx]

**A system dynamics model for pests and natural enemies interactions**

**Bonoukpoè Mawuko Sokame^1^; Henri E.Z. Tonnang^1*^; Sevgan Subramanian^1^; Anani Y. Bruce^2^; Thomas Dubois^1^; Sunday Ekesi^1^ and Paul-André Calatayud^1,3^**

^1^International Centre of Insect Physiology and Ecology (*icipe*), P.O. Box 30772-00100, Nairobi, Kenya

^2^International Maize and Wheat Improvement Center (CIMMYT) ICRAF House, United Nation, Avenue, Gigiri, P. O. Box 1041 Village Market, Nairobi 00621, Kenya

^3^IRD, CNRS, Université Paris-Saclay, UMR Évolution, Génomes, Comportement et Écologie, 91198, Gif-sur-Yvette, France

^*^Corresponding author:

Henri E.Z. Tonnang

[htonnang@icipe.org](mailto:htonnang@icipe.org)

**Appendix: Model notations and equations**

**Table:** Notations used for developed models

| **Notation** | **Variable name** | **Description** | **Units** |
| --- | --- | --- | --- |
| PSi | Pest species i | Population of pest species i susceptible to parasitism by parasitoids and competitive interactions with other pest species j in the community | Pest |
| APPSi | Associated Parasitoids of PSi | Population of parasitoid species that parasitized the pest species i population in the community | Parasitoid |
| Ki | Carrying Capacity of PSi | Represent the maximum number of Psi individuals that one hectare of maize field can support | Pest |
| NPi | Number of parasitoids per host | Represent average number of parasitoids that one cocoon mass from one parasitized host | Parasitoid |
| FPi | Female Parasitoid i | Part of parasitoid population for parasitism | Parasitoid |
| PSiGR (t) | PSi Growth Rate | Increase rate of pest species i per month | Pest/Month |
| PSiDR (t) | Psi Decrease Rate | Rate of decrease in pest species i population from parasitism | Pest/Month |
| PSiPGR (t) | PSi Parasitoids Growth Rate | Increase rate of PSi associated parasitoids from parasitism | Parasitoid/Month |
| PSiPDR (t) | PSi Parasitoids Decrease Rate | Natural rate of decrease of parasitoids associated of Psi from mortality and emigration. | Parasitoid/Month |
| PSiMP | PSi Meeting Probability | Probability that the female parasitoid meet the host in the field for parasitism | Pest/Parasitoid/  Month |
| d(PSis) | Average time to start the evaluation of PSi | Average duration for maize plants to get infested after planting date | Month |
| d(PSip) | Average time to start recording parasitism of PSi | Average duration for maize plants to recruit parasitoids for defence after planting date | Month |
| PSiFGR (t) | PSi Fractional Growth Rate | Fractional rate of increase of pest species i | Dmnl/Month |
| PSiRFGR (t) | PSi Reference Fractional Growth Rate | Maximum fractional growth rate reported of pest species I in the literature | Dmnl/Month |
| PSiFPR (t) | PSi Fractional Parasitism Rate | Fractional rate of increase of parasitoid species i | Dmnl/Month |
| PSiRFPR | PSi Reference Fractional Parasitism Rate | Parasitism in maize field (Pm) and Parasitism in wild habitat (Pw) | Dmnl/Month |
| PiFDR (t) | Parasitoid i Fractional Decrease Rate | Fractional rate of decrease of pest species i associated parasitoids | Dmnl/Month |
| aij | Competition coefficient | Represent the effect of an individual of the j^th^ species on the i^th^ species | Dmnl/Pest |
| PSiHP | Host plant for PSi | Total number of host plant in maize filed (Hm) and in wild habitat during off-seasons (Hw) | Plant |
| PSiRD | PSi Reference Density | Maximum pest density reported in the literature | Pest/Plant |
| SRi | Sex Ratio i | Sex ratio of a given parasitoid species | Dmnl |
| β | Intra-specific interaction effect | Proportion of pest present in the field in relationship to the carrying capacity | Dmnl |
| δ | Inter-specific interaction effect | Represent the effect that other species PSj exert on PSi in multi-species community | Dmnl |
| λi(t) | Discrepancy between PSi&Ki | Proportional gap between population of pest species i and its carry capacity | Dmnl |
| Nj | Total population of Pest j | Total population of Pest j | Pest |

**Equations:**

$$\frac{d\mathrm{PSi}}{dt}=PSiGR (t)-PSiDR (t) (1)$$

$$\frac{d\mathrm{APPSi}}{dt}=\mathrm{PSiPGR}\left( t \right)-\mathrm{PSiPDR}\left( t \right) (2)$$

$$\mathrm{PSiGR}\left( t \right)=\mathrm{PSiFGR}\left( t \right)˟ \frac{d\mathrm{PSi}}{dt} (3)$$

$$\mathrm{PSiDR}\left( t \right)=\mathrm{PSiFPR}\left( t \right)˟ \frac{d\mathrm{PSi}}{dt} (4)$$

$$\mathrm{PSiPGR}\left( t \right)=\mathrm{PSiDR}\left( t \right)˟ NPi (5)$$

$$\mathrm{PSiPDR}\left( t \right)=\mathrm{PSiRD}˟ \frac{d\mathrm{APPSi}}{dt} (6)$$

$$\mathrm{PSiHP}=\mathrm{MAX}\left( Hw, Hm*PULSE TRAIN\left( 3, 3 , 6 , 24 \right) \right) (7)$$

$$\mathrm{Ki}=\mathrm{PSiDR}˟ PSiHP (8)$$

$$\beta i =\frac{\int_{0}^{t} \frac{ⅆ\mathrm{PSi}}{\mathrm{dt}}}{\mathrm{Ki}} (9)$$

With Lookup: ((0,0), (0.12,0.5), (0.25,1.2), (0.75,1.4), (0.5,1.6), (1,1))

$$\delta i=\sum_{k=1}^{m} \mathrm{aijNj} (10)$$

$$\mathrm{Nj}=\int_{0}^{t} \frac{ⅆPSj}{dt} (11)$$

$$\lambda i\left( t \right)=1-\left( \beta i+ \delta i \right) (12)$$

$$\mathrm{PSiFGR}\left( t \right)=\mathrm{SMOOTH}\left( \lambda i\left( t \right)˟ \mathrm{PSiRFGR}\left( t \right), d\left( \mathrm{PSis} \right) \right) (13)$$

$$\mathrm{FPi}\left( t \right)=\mathrm{SRi}˟ \frac{d\mathrm{APPSi}}{dt} (14)$$

$$\mathrm{PSiRFPR}=\mathrm{MAX}\left( Pw,Pm*PULSE TRAIN\left( 3, 3 , 6 , 24 \right) \right) (15)$$

$$\mathrm{PSiFPR}\left( t \right)=DELAY3\left( \mathrm{MAX}\left( \mathrm{PSiRFPR}, \left( \mathrm{PSiMP}\mathrm{SRi}˟ \frac{\mathrm{FPi}\left( t \right)}{\frac{d\mathrm{PSi}}{dt}} \right) \right),d\left( \mathrm{PSip} \right) \right) (16)$$
